# The protective effect of *Gentiana lutea* flower against mycotoxin toxicity in a gastrointestinal barrier *in vitro* model

**DOI:** 10.3389/fnut.2025.1627476

**Published:** 2025-08-12

**Authors:** Giacomo Di Matteo, Massimo Frangiamone, Pilar Vila-Donat, Valter Di Cecco, Luciano Di Martino, Lara Manyes, Luisa Mannina

**Affiliations:** ^1^Food Chemistry Lab, Department of Chemistry and Technology of Drugs, Sapienza University of Rome, Rome, Italy; ^2^NMR-Based Metabolomics Laboratory of Sapienza (NMLab), Sapienza University of Rome, Rome, Italy; ^3^Fondazione OnFoods, Parma, Italy; ^4^Department of Biomedical Sciences, University of Lausanne, Lausanne, Switzerland; ^5^Laboratory of Food Chemistry and Toxicology, Faculty of Pharmacy, Universitat de València, Valencia, Spain; ^6^Maiella Seed Bank, Maiella National Park, Sulmona, Italy

**Keywords:** mycotoxin, *Gentiana lutea*, qPCR, *in vitro* digestion, metabolomics, NMR, HPLC, bioactive compounds

## Abstract

**Objective:**

We investigated the phytochemical composition, gastrointestinal bioaccessibility, and protective effects against mycotoxin-induced toxicity of *Gentiana lutea* L. flower, a botanical species traditionally used in European herbal medicine.

**Methods:**

*Gentiana lutea* flower samples were collected from the Majella National Park and chemically characterized using HPLC-PDA and NMR metabolomics, revealing the presence of abundant bioactive compounds (iridoids, secoiridoids, and xanthones), as well as quantifying the levels of amino acids, organic acids, and sugars. Following *in vitro* gastrointestinal digestion, bioaccessible fractions were analyzed and subjected to transepithelial transport assays using differentiated Caco-2 monolayers.

**Results:**

Gene expression analysis and cytotoxicity evaluation on Caco-2 cell cultures demonstrated that digested *Gentiana* significantly mitigated the toxic effects of aflatoxin B1 (AFB1), ochratoxin A (OTA), and beauvericin (BEA). The digested *Gentiana* samples reduced the expression of pro-apoptotic genes (BAX, CASP3), preserved intestinal barrier integrity by modulating tight junction-related genes (CL-2, ZO-1), and promoted antioxidant responses through SRXN1 regulation.

**Conclusion:**

These findings highlight the potential of *Gentiana lutea* flowers as a source of functional phytocompounds for intestinal barrier protection against mycotoxins.

## 1 Introduction

Mycotoxins are toxic secondary metabolites produced by various filamentous fungi, primarily from the genera *Aspergillus, Penicillium*, and *Fusarium*, which are ubiquitous contaminants in food and feed. Once ingested, these compounds may provoke a wide range of toxic effects, including hepatotoxicity, nephrotoxicity, neurotoxicity, immunosuppression, and carcinogenicity. Among them, aflatoxin B1 (AFB1) and ochratoxin A (OTA) have proven not only to be the most toxic and extensively studied but also to have captured the attention of the scientific community. In detail, AFB1, produced mainly by *Aspergillus flavus* and *A. parasiticus*, is highly hepatotoxic and carcinogenic, whose mode of action involves the production of a reactive epoxide that binds to DNA, leading to mutations and tumorigenesis (1). OTA, primarily produced by *Aspergillus* and *Penicillium* species, disrupts protein synthesis and induces oxidative stress, contributing to nephrotoxicity and immunosuppression (2). Other emerging mycotoxins, such as beauvericin (BEA), a cyclohexadepsipeptide produced by *Fusarium* species, exhibit ionophoric properties, disturbing cellular ion homeostasis and inducing apoptosis (3). In this context, it is essential to emphasize that the gastrointestinal tract is the primary site of exposure to mycotoxins, which can compromise the gut barrier integrity, leading to increased intestinal permeability and subsequent systemic toxin dissemination. Therefore, understanding the mechanisms by which mycotoxins disrupt the gastrointestinal barrier, as well as identifying potential protective strategies, is essential for protecting human and animal health and for developing effective interventions (4–6). In recent years, there has been a growing interest in the use of natural products, particularly those derived from medicinal plants, as potential protective agents against mycotoxin-induced toxicity (7). Among these plants, *Gentiana lutea*, a perennial herbaceous plant native to mountainous regions of Europe, has garnered attention due to its rich phytochemical profile and the traditional use of its roots in European herbal medicine for treating digestive disorders, liver ailments, and inflammation (8–11). *Gentiana lutea* rootcontains a variety of bioactive compounds, including iridoids, secoiridoids, xanthones, flavonoids, and phenolic acids (12–15). However, the wild harvesting of roots has led to a depletion of this species in its natural habitats (16), and there is growing interest in studying its aerial parts (17) as they are rich in the same bioactive compounds and are renewable resources that can be used without damaging the plant. These compounds from the *Gentiana* genus have been shown to exhibit multiple pharmacological activities, including antioxidant, anti-inflammatory, and gastroprotective effects (18). The iridoids and xanthones, in particular, are believed to contribute significantly to the plant's protective effects by scavenging free radicals, modulating inflammatory pathways, and enhancing the integrity of cellular membranes (7, 19, 20). Moreover, *Gentiana lutea* flower was reported to counteract BEA-induced cytotoxicity in Jurkat cells in a proteomics study (21). The protective effects of *Gentiana lutea* against gastrointestinal toxicity are of particular interest, especially in the context of mycotoxin exposure. Previous studies have demonstrated that extracts from *Gentiana lutea* can mitigate reactive oxygen species formation and inflammation in various *in vitro* and *in vivo* models, suggesting a potential role in protecting the GI barrier from mycotoxin-induced damage (22). However, the precise molecular mechanisms underlying this protective role remain largely unexplored.

In the present study, the molecular mechanisms by which *Gentiana lutea* exerts its beneficial effects in response to gastrointestinal toxicity induced by mycotoxin exposure were investigated. In particular, *Gentiana lutea* flower samples were first chemically characterized using NMR-based metabolomics and HPLC-PDA. Then, in order to simulate physiologically relevant exposure conditions, the combined use of *in vitro* digestion and Caco-2 cell cultures was performed. Caco-2 cells were differentiated for 21 days, to mimic the intestinal epithelial component of the gastrointestinal barrier *in vitro*, and exposed to AFB1, OTA, and BEA individually and in combination with the digested *Gentiana* flower. Gene expression analysis was performed to identify specific genes and signaling pathways that are modulated by *Gentiana lutea* in response to mycotoxin exposure, providing insights into the cellular processes involved in maintaining barrier integrity and mitigating oxidative stress and apoptosis (23). In addition, the bioaccessibility and the transepithelial transport of the main costituents of *Gentiana* flowers was evaluated by both NMR and HPLC-PDA analysis.

## 2 Material and methods

### 2.1 Reagents

Methanol and dimethyl sulfoxide were supplied by Sigma-Aldrich (St Louis, USA). The reagents used for cell culture, such as Dulbecco's Modified Eagle's Medium (DMEM), penicillin, fungizone, streptomycin, non-essential amino acids (NEAA), HEPES (4-(2-hydroxyethyl)-1-piperazineethane-sulfonic acid), phosphate-buffered saline (PBS), Hank's balanced salt solution (HBSS), fetal bovine serum (FBS) and trypsin-EDTA were purchased from Thermo Fisher Scientific (MA, USA). Milli-Q H_2_O (< 18 MU cm resistivity) was obtained from a Milli-QSP^®^ reagent water system (Millipore, Bedford, MA, USA). Standard mycotoxins: AFB1 (MW: 312.28 g/mol), OTA (MW: 403.81 g/mol), and BEA (MW: 783.95 g/mol) were obtained from Sigma-Aldrich (St. Louis, MO), and stock solutions were dissolved in methanol at 1 mg/ml and maintained at −20 °C. Gastrointestinal digestion reagents, including formic acid, hydrochloric acid, monosodium phosphate, potassium chloride, potassium thiocyanate, sodium chloride, sodium bicarbonate, sodium sulfate, sodium hydroxide, as well as α-amylase, bile salts, pancreatin, pepsin, and urea, were supplied by Thermo Fisher Scientific (MA, USA). 3-(4,5-Dimethylthiazol-2-yl)-2,5-diphenyltetrazolium bromide (MTT) and phenol red were obtained from Sigma-Aldrich (St Louis, USA). For transcriptomic analysis, the ReliaPrep™ RNA Cell Miniprep System Kit was purchased from Promega (WI, USA), while the TaqMan Reverse Transcription Kit and SYBR Green PCR Master Mix were from Thermo Fisher Scientific. The iridoid, secoiridoid, and xanthone standard compounds: loganic acid, swertiamarin, gentiopicroside, sweroside, mangiferin, amarogentin, and isogentisin were purchased from Merck Life Science (Milan, Italy). Acetonitrile LC-MS grade was purchased from Fisher Scientific (MA, USA).

### 2.2 *Gentiana lutea* flower collection

The flowers of wild *Gentiana lutea* subsp. *lutea* (*G. lutea*) were collected in the Maiella National Park (740 km^2^; 130–2,793 m asl, Central Apennines, Italy) in July 2021. The population of *G. lutea* subsp. Lutea was found in a clearing of a mountain pine forest (*Pinus mugo*) extending over an area of ~50 m^2^ in “Monte Cavallo” locality at 2,100 m asl (42°7′29.42″N; 14°6′50.11″E). The botanical origin was identified by Dr. Luciano Di Martino and Dr. Valter Di Cecco of the Majella National Park. The flowers were freeze-dried, blended, and then stored at −20 °C until analysis.

### 2.3 Methanolic extraction of iridoids, secoiridoids, and xanthones

An extraction with methanol was considered the most effective extraction method for the quantitative isolation of the main bioactive compounds (iridoids, secoiridoids, and xanthones) of the *Gentiana* plant (24). Hence, 0.100 g of *Gentiana* dry flowers was extracted with 3 ml of methanol (rate of 1:30 w/v) using a 20 min ultrasonic bath (Ultrasonic cleaner, VWR, Milan, Italy) at room temperature. Then, the pellet and extract were separated by centrifugation (Centrifuge 5810 R, Eppendorf, Milan, Italy) at 4,000 rpm for 5 min, and the supernatant was collected. The extraction procedure was repeated two more times on the same pellet, and the supernatants were collected together, yielding the *Gentiana* flower methanol extract (GME). The entire process was performed in triplicate, and the extracts were analyzed or frozen until their use (−20 °C).

### 2.4 *In vitro* human digestion

A static *in vitro* gastrointestinal digestion model was used to reproduce the physiological process of human digestion based on a previously reported protocol (25). Briefly, to simulate the oral phase, 600 μl of prepared artificial saliva and 8.4 ml of water were added to 100 mg of *Gentiana* flower and incubated for 2 min at 37 °C in the dark with gentle agitation in an orbital shaker (100 rpm; Optic evymen system, Spain). Then 0.5 g of pepsin solution (0.040 g/ml pepsin in 0.1 N HCl) was added, the pH was adjusted to 3 with 6 N HCl, and the samples were incubated for 2 h at 37 °C in the dark with gentle agitation to obtain the gastric digests of *Gentiana*. After the incubation time, the pH was adjusted to 7 with 1 N NaHCO_3_ and 1.25 g of bile salts/pancreatin mixture (4.0 mg/ml pancreatin and 25 mg/ml bile salts in 0.1 N NaHCO_3_) was added. The samples were incubated again for 2 h at 37 °C in the dark with gentle agitation. At the end, the samples were centrifuged (4,000 rpm for 5 min at 4 °C), obtaining the intestinal digests of *Gentiana* that were analyzed or frozen until their use (−80 °C). All digestions were performed in triplicate, obtaining three biological replicated of both the *Gentiana* flower gastric phase (GGP) and *Gentiana* flower duodenal phase (GDP). A blank sample, substituting the sample with the same amount of water, was also realized and chemically analyzed. Bioaccessibility of *Gentiana* bioactive compounds was calculated as the percentage of compounds from the methanol extraction (GME), considered a quantitative extraction method for iridoids, secoiridoids, and xanthones, that were detected in the digested extracts (GDP and GGP) using the following equation.


$$\begin{array}{l} Bioaccessibility\;\left(\%\right)=\frac{\left[Digested\;extract\right]}{\left[Methanol\;extract\right]}\times \;100 \end{array}$$

### 2.5 Cell culture

Caco-2 cells were chosen as suitable model to mimic the intestinal epithelial component of the gastrointestinal barrier *in vitro*. Caco-2 cells were cultured in DMEM medium supplemented with 100 U/ml penicillin, 100 mg/ml streptomycin, 10% FBS, 1% NEAA, and 1% HEPES. The incubation conditions were pH 7.4, 5% CO_2_ at 37 °C and 95% air atmosphere at constant humidity, maintained using a Thermo Forma Steri-Cycle CO_2_ incubator (Thermo Scientific, MA, USA). To maintain an optimal viability level, medium was changed every 2–3 days. In order to reproduce the gastrointestinal *in vitro* barrier, Caco-2 cells were consistently differentiated for 21 days on well plate prior to use in all experiments.

### 2.6 Cell viability analysis

MTT was used to assess the viability of differentiated Caco-2 cells after exposure to different dilutions of GDP. The assay was performed according to the protocol described by Frangiamone et al. (26) with some caveats. Cells were plated in 24-well microplates at a density of 5 × 10^4^ cells/well. After 21 days of differentiation, the culture medium was replaced with a fresh medium containing five serial dilutions from 1:1 to 1:16 (dilution factor = 2) of GDP. After 4, 24, 48, and 72 h incubation, the medium with the GDP was replaced with 250 μl of fresh medium containing 50 μl of MTT solution (0.5 mg/ml). After 4 h of incubation (37 °C in the dark), 400 μl of DMSO was added and cytotoxicity was determined. Absorbance was measured at 620 nm using a Wallace Victor2 multi-label counter, model 1420 (Perkin Elmer Life Sciences, Waltham, MA, USA). Cell viability was expressed as percentage relative to the control (cells and medium). All experiments were performed in quadruplicate with technical replicates.

### 2.7 *In vitro* transepithelial transport evaluation

Transepithelial transport of *Gentiana* bioactive compounds in GDP was evaluated using differentiated Caco-2 cells. To reproduce the intestinal barrier, Caco-2 cells were seeded at a density of 2.5 × 10^5^ cells per well in 6-well polycarbonate plates with Transwell^®^ permeable media, having a diameter of 23 mm and a pore size of 0.4 μm (Corning Life Sciences, USA). Cells were cultured for 21 days until complete differentiation, renewing the medium bilaterally every 2–3 days. On day 21, the integrity of the intestinal monolayer was assessed using a phenol red permeability assay (*n* = 3), where cell passage of < 6% indicated adequate monolayer formation. Briefly, cells were washed three times with PBS and incubated for 1 h with 0.6 ml of phenol red (42 μM) in the apical compartment and 1.5 ml of PBS in the basolateral compartment. Phenol red transition was determined by measuring absorbance at 558 nm (UV-1600PC Spectrophotometer) after pH adjustment to 11 (25). In addition, barrier integrity was also assessed by measuring transepithelial electrical resistance, both before and after the transport assay. Values above 400 Ω·cm^2^ are considered indicative of a functionally intact gut epithelial barrier. Once optimal barrier integrity was confirmed, the differentiated cells were washed twice with HBSS (transport medium). Then, 2 ml of HBSS was added to the basolateral compartment (representative of the bloodstream) and 600 μl of GDP was added to the apical compartment (representative of the intestinal lumen). Then, the basolateral compartments were collected after 4 h of incubation at 37 °C for HPLC analysis. The assay was repeated on six different wells. Transepithelial transport was expressed as the percentage of the studied bioactive compounds from the duodenal digest added in the apical compartment (0 h) that reached the basolateral compartment after 4 h of exposure. In addition,


$$\begin{array}{l} Transepithelial\;transport\;\left(\%\right)=\frac{\left[Basolateral\;4\;h\right]}{\left[Duodenal\;digested\right]}\times \;100 \end{array}$$

### 2.8 HPLC-PDA analysis

HPLC-PDA analysis was carried out to identify and quantify the main bioactive compounds of the *Gentiana* genus in the methanolic extract (GME), in the two digested extracts (GGP and GDP), and in the basolateral compartment of transepithelial transport assay. The chromatographic analysis was performed with an HPLC instrument (Agilent 1100 series, Waldbronn, Germany) equipped with a PDA detector, column oven, quaternary pump, degasser, and autosampler. The separation was performed with a Kinetex EVO C18 column (4.6 mm × 150 mm × 5 μm, Phenomenex, Bologna, Italy), a flow of 1.0 ml/min, and a binary mobile phase consisted of water/formic acid 99.9/0.1 % v/v (A) and acetonitrile (B) combined in a gradient as follows: 0–5 min, 5%−5% B; 5–30 min 5%−30% B; 30–34 min 30%−41% B; 34–37 min 41%−57% B; 37–39 min 57%−57% B; 39–48 min 57%−100%; 48–50 min 100%−100%; 50–55 min 100%−5%; 55–65 min 5%−5% B. The samples of *Gentiana* flower methanol extract (GME), duodenal and gastric phases (GDP and GGP), basolateral compartment, and mix of commercial standards were filtered through a 0.22 μm nylon syringe filter (Membrane Solutions, Shanghai, China), and 5 μl was directly injected into the column pre-heated at 30 °C. Separations were monitored at 258 nm, and the peaks were integrated using the OpenLab ChemStation software (Agilent, Waldbronn, Germany). Quantification was performed using the external standard method, generating a calibration curve with commercial standards ranging from 0.5 to 50 μg/ml with 7 points. The results are expressed as mg/g of dry weight (DW) ± standard deviation (SD).

### 2.9 NMR analysis

Both GGP and GDP were analyzed by metabolomics NMR to identify and quantify the levels of amino acids, organic acids, and sugars. In particular, the GGP and GDP samples were freeze-dried and resuspended in 1 ml of a solution containing 400 mM phosphate buffer/D_2_O, 0.200 mM 3-(trimethylsilyl)-propionic-2,2,3,3-d4 acid sodium salt (TSPA), and EDTA-d16. Then, the solution was centrifuged, and 0.7 ml was transferred into a 5 mm NMR tube. The NMR spectra were recorded at 25 °C on a JNM-ECZ 600R (JEOL Ltd., Tokyo, Japan) spectrometer operating at the proton frequency of 600.17 MHz equipped with an autosampler. The ^1^H spectra of the digested samples were acquired using a presaturation pulse sequence to suppress the water signal, a 90° pulse of 8.3 μs, 256 transients, and 65 K data points. All the NMR spectra were processed using the JEOL Delta v5.3.1. software (JEOL Ltd, Tokyo, Japan). The ^1^H NMR spectra were Fourier transformed, manually phased, automatically base corrected, and referred to the internal standard TSPA set at 0.00 ppm. Metabolite assignments were performed by previous NMR metabolomics studies (27–29) and by 2D NMR experiments, namely ^1^H-^1^H TOCSY, ^1^H-^13^C HSQC, and ^1^H-^13^C HMBC. In order to absolute quantify the assigned metabolites in the analyzed aqueous samples (GGP and GDP), the integral of the corresponding selected ^1^H resonances (reported in Supplementary Table S1) were measured with respect to the integral of the TSP methyl group signal normalized to 100, and the quantitative results were expressed as mg/100 g of DW ± SD.

### 2.10 qPCR analysis

For qPCR analysis the differentiated Caco-2 cells were exposed to AFB1, OTA and BEA individually and in combination among them (100 nM in all cases) or combined with GDP (1:1 as dilution) during 4 h. First, total RNA was isolated from control and exposed Caco-2 cells using the ReliaPrep™ RNA System Kit and purified to exclude DNA contamination. Then, RNA extracted from each sample was checked for quantity and quality using a NeoDot UV/Vis Nano Spectrophotometer (Neo Biotech, Nanterre, France), showing appropriate 260/280 nm and 260/230 nm ratios both around 2. RNA samples were stored at −20 °C until they were diluted to 100 ng/μl with sterilized Milli-Q H_2_O. After that, cDNA was synthesized using the TaqMan Reverse Transcription Kit for qPCR analysis.

Gene-specific primers were designed using Primer-BLAST, with amplified products ranging from 65 to 150 bp and melting temperatures (Tm) between 58 and 60 °C. For some genes, such as ZO-1, sulfiredoxin 1 (SRXN1), and s18, primer sequences were adopted from previously published studies (30, 31). All primers were validated in the present study by generating standard curves from five-fold serial dilutions of cDNA. Melting curve analysis performed on the StepOne Plus Real-Time PCR system (Applied Biosystems, CA, USA) confirmed the presence of a single amplification product for each gene. The correlation coefficient (*R*^2^, indicating linearity) and amplification efficiency (E) for each primer pair were calculated from the slope of the regression line plotting the mean Cq values against the log dilution factor of cDNA using StepOne software. The gene-specific primers used in this study are listed in Table 1.

**Table 1:** Gene names, forward and reverse primer sequences, amplification efficiency, and linearity value for the target genes and the reference gene β-actin.

**Gene**	**Primer forward sequence**	**Primer reverse sequence**	***E* (%)**	***R* ^2^**
18S rRNA	CGGCTACCACATCCAAGGAA	GCTGGAATTACCGCGGCT	105	0.991
BAX	ATGCGTTTTCCTTACGTGTCT	GAGGTCAGCAGGGTAGATGA	105	0.983
CASP-3	GGAGGCCGACTTCTTGTATG	GCCATCCTTTGAATTTCGCC	107	0.984
CL-2	CTCCCTGGCCTGCATTATCTC	ACCTGCTACCGCCACTCTGT	116	0.983
ZO-1	CAACATACAGTGACGCTTCACA	CACTATTGACGTTTCCCCACTC	121	0.990
SRXN1	GGTCTAGGGGAAGAGGTGTT	CTTGGTTTTCAGAAGCCCCT	109	0.992

Real-time amplification reactions were performed in 96-well plates using SYBR Green dye and were run in triplicate on 96-well plates with a StepOne Plus Real-time PCR instrument (Applied Biosystems, Foster City, CA, USA). Reactions were prepared as follows: 100 ng of template, 500 nM of each primer, the required amount of 2 × Fast SYBR Green, and brought to a total volume of 20 μl with RNAse-free water (Applied Biosystems, Foster City, CA, USA). Cycling conditions for apoptosis-related genes were set as the default: initial denaturation step at 95 °C for 5 min to activate Taq DNA polymerase, followed by 45 cycles of denaturation at 95 °C for 30 s, annealing at 58 °C for 30 s, and elongation at 72 °C for 40 s. For barrier integrity-related genes, the cycling conditions are as follows: an initial denaturation step at 95 °C for 5 min to activate Taq DNA polymerase, followed by 40 cycles of denaturation at 95 °C for 10 s and annealing at 60 °C for 30 s. For SRXN1, the qPCR instrument was set to the following conditions: an initial denaturation step at 95 °C for 5 min to activate Taq DNA polymerase, followed by 40 cycles of denaturation at 95 °C for 15 s, annealing at 58 °C for 15 s, and elongation at 72 °C for 45 s. The melting curve was generated by heating the amplicon from 60 to 90 °C. Threshold cycles (Ct) were determined using StepOne Plus version 2.3 software, and data were analyzed using the 2^ΔΔCt^ method. Ribosomal s18 was used as a housekeeping gene, whose expression remained stable under treatment conditions. Three technical replicates were performed for each condition. The experiments were conducted in accordance with the MIQE (Minimum Information for Publication of Quantitative Real-Time PCR Experiments) guidelines (32). To evaluate the statistical analysis, the ΔCt (experimental Ct – mean of maintenance Ct) obtained by qPCR was used. Levene's test was used to assess the equality of variances between groups, and the T-student test was applied to assess differences between groups. For statistically significant differences, *p* ≤ 0.05 was considered.

### 2.11 Statistical analysis

All quantitative data, including qPCR (after ΔCt transformation), MTT cell viability assays, HPLC, and NMR results, were tested for normality using the Shapiro–Wilk test and for homoscedasticity using Levene's test in GraphPad Prism (version 10). Since the data met the assumptions of normality and equal variances, parametric statistical tests were applied: student's *t*-test for pairwise comparisons and one-way ANOVA, followed by Tukey's *post hoc* test for comparisons involving more than two groups. Statistical significance was set at *p* ≤ 0.05.

## 3 Results and discussion

### 3.1 Bioactive compounds in *Gentiana lutea* flower

**Figure 1:**
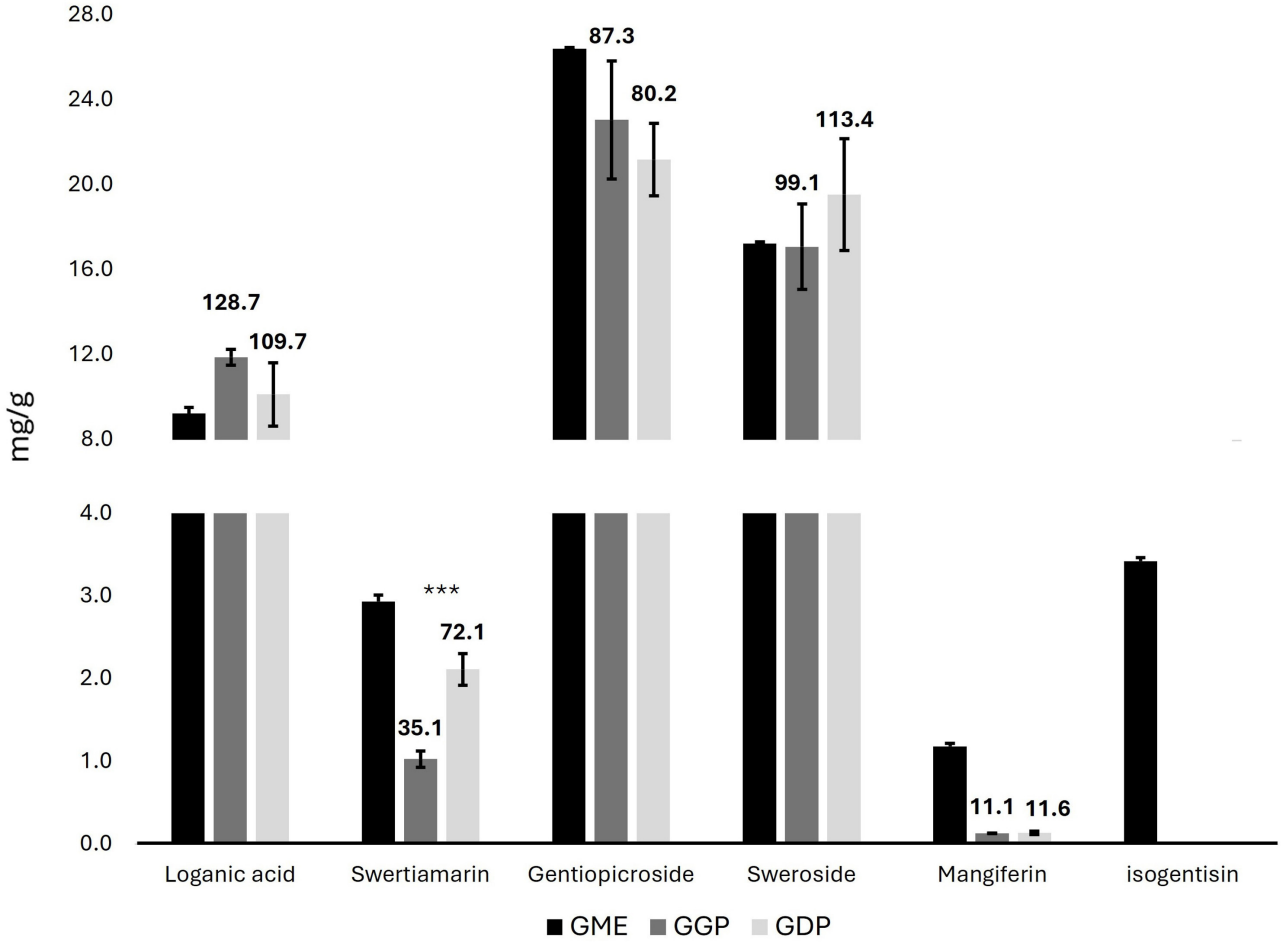
Concentrations of iridoids, secoiridoid, and xanthone compounds in *G. lutea* flowers methanol extract, gastric phase, and duodenal phase in mg/g of DW ± SD by HPLC-PDA. Data above bars express the bioaccessibility of each compound (GME concentration representing 100% of bioaccessibility). GME, *Gentiana* flower methanol extract; GGP, *Gentiana* flower gastric phase; GDP, *Gentiana* flower duodenal phase. *p* < 0.05; **p < 0.01, ***p < 0.001 significant differences among GGP and GDP.

The bioactive compounds of *G. lutea* flowers were determined by targeted HPLC-PDA analysis. In particular, the chromatographic analysis of the different *G. lutea* flower preparations was performed to identify and quantify the iridoid, secoiridoid, and xanthone contents, in which the methanol extract (GME) is used as a quantitative measure of the bioactive compound content in *G. lutea* flowers. The chromatogram of GME was reported in Supplementary Figure S1. In particular, the levels of one iridoid (loganic acid), three secoiridoids (sweroside, swertiamarin, and gentiopicroside), and two xanthones (mangiferin, isogentisin) were determined in GME. The identification was carried out by comparing their retention times and UV spectra with the external standards. Quantification was based on the external standard method, and the results were expressed as mg/g of DW ± SD (Figure 1). A marogentin, another characteristic secoiridoid compound of *G. lutea* roots, was not detected in the flowers (33). Menković et al. have analyzed *G. lutea* flowers harvested at four different natural localities, finding a high presence of secoiridoid and xanthone compounds. Gentiopicroside was the main secondary metabolite in all the analyzed *G. lutea* flowers of Menković et al., and the same was found in our *G. lutea* flower from Majella National Park of Italy at the same level. Gentiopicroside has demonstrated anti-inflammatory and gastroprotective properties in several *in vitro* and *in vivo* studies, which may be highly relevant for counteracting the harmful effects of mycotoxins on the gastrointestinal tract. In particular, the key anti-inflammatory targets of gentiopicroside include the inhibition of iNOS, COX-2, and pro-inflammatory cytokines (TNF-α, IL-1β, IL-6, and IL-8), alongside the restoration of IL-10 levels. These effects are mediated through the suppression of the NF-κB and p38MAPK pathways, as well as the inhibition of NLRP3 inflammasome activation (34, 35). Swertiamarin, another secoiridoid compound, was not detected in the flowers of Menković et al. However, in the present study, it was detected with a concentration of 2.93 mg/g. Swertiamarin meets all five of Lipinski's rules for drug-like properties and is studied for numerous activities, including gastroprotective, anti-inflammatory and antioxidant, The gastroprotective activity of swertiamarin is probably due to the inhibition of the dopamine D2 receptor; while the anti-inflammatory activity to the reduction of pro-inflammatory cytokines such as TNF-α, IL-6, IL-1β, and IL-8, and the increased expression of anti-inflammatory cytokines, including IL-4 and IL-10 (36). Moreover, swertiamarin shows antioxidant activity by reducing oxidative stress and apoptosis-related markers such as caspase-3 and PARP1. It also regulates AMPK and suppresses PEPCK, indicating a role in maintaining metabolic balance. Regarding mangiferin, the levels reported in the article were at least seven times higher than those in the present study, whereas the quantity of isogentisin, another xanthone compound, was comparable (37). Similarly, Niu et al. have analyzed the changes in secondary metabolites of *Gentiana macrophylla*, another plant of the *Gentiana* genus, during flower development (38). Mangiferin has shown a gastroprotective effect by modulating inflammation, oxidative stress, and apoptosis, possibly through the PPAR-γ/NF-κB and Nrf2/heme oxygenase-1 (HO-1) signaling pathways. Moreover, mangiferin helps preserve intestinal barrier integrity by modulating tight junction proteins such as occludin, and it has shown protective effects in inflammatory bowel disease models by reducing inflammation and restoring barrier function (39). Finally, our loganic acid level was comparable to the levels in the initial stage of the seed-producing period reported in the article, while the levels of sweroside in our *G. lutea* were higher. Loganic acid has been shown to reduce the expression of COX-2 and iNOS in LPS-stimulated J774A.1 macrophages, confirming its anti-inflammatory potential *in vitro* (40).

### 3.2 Bioaccessibility assessment

Bioaccessibility refers to the ability of bioactive compounds to be released from their food matrix during digestion, and it represents a key factor influencing their subsequent biological potential through absorption (bioavailability). The two digested extracts (GGP and GDP) were analyzed using both targeted HPLC-PDA analysis to determine the bioactive compounds and metabolomics NMR to determine the concentrations of sugars, organic acids, and amino acids. In particular, the HPLC-PDA analysis of GGP and GDP was carried out to calculate the bioaccessibility of the iridoids, secoiridoids, and xanthones of *G. lutea* flowers. The chromatograms are reported in Supplementary Figure S2. All the determined bioactive compounds, except for swertiamarin, were completely released from the matrix in the gastric phase, and a similar amount was found in the intestinal phase. Loganic acid and sweroside had the highest percentage of bioaccessibility, whereas mangiferin had the lowest. Loganic acid release from olive leaf has been previously studied by Duque-Soto and coworkers, yielding intestinal bioaccessibilities of 68.43% and 39.40% for the two loganic acid isomers (41). The lower loganic acid bioaccessibility could be associated with the specific interactions of iridoids within the olive leaf matrix, in contrast to the high percentage of release obtained from the *Gentiana* flower matrix. Regarding swertiamarin, its level increased during the digestion from the gastric to the intestinal phase, resulting in a doubling of its release from the matrix. The *in vitro* bioaccessibility of mangiferin has been previously studied by Herrera-Cazares et al., obtaining a higher intestinal release from mango bagasse (42). Our low bioaccessibility percentage of mangiferin aligns with the literature, which indicates its limited intestinal absorption due to poor aqueous solubility. In particular, Herrera-Cazares and coworkers reported that interactions with food components, such as lipids, can enhance mangiferin dissolution and absorption, which may be lacking in our matrix. Additionally, matrix-related effects may have limited its release or promoted efflux mechanisms, further reducing its intestinal availability (42). To enhance its absorption and biological activity, various delivery systems and structural modifications have been explored, including nanoemulsions, nanoparticles, gelation techniques, solid dispersions, and the synthesis of analogs or derivatives. These strategies have shown promising results, with several studies reporting a notable improvement in the anti-inflammatory activity of modified mangiferin formulations (39). Since isogentisin was not detected in the bioaccessible fractions, its bioaccessibility was not calculated.

**Figure 2:**
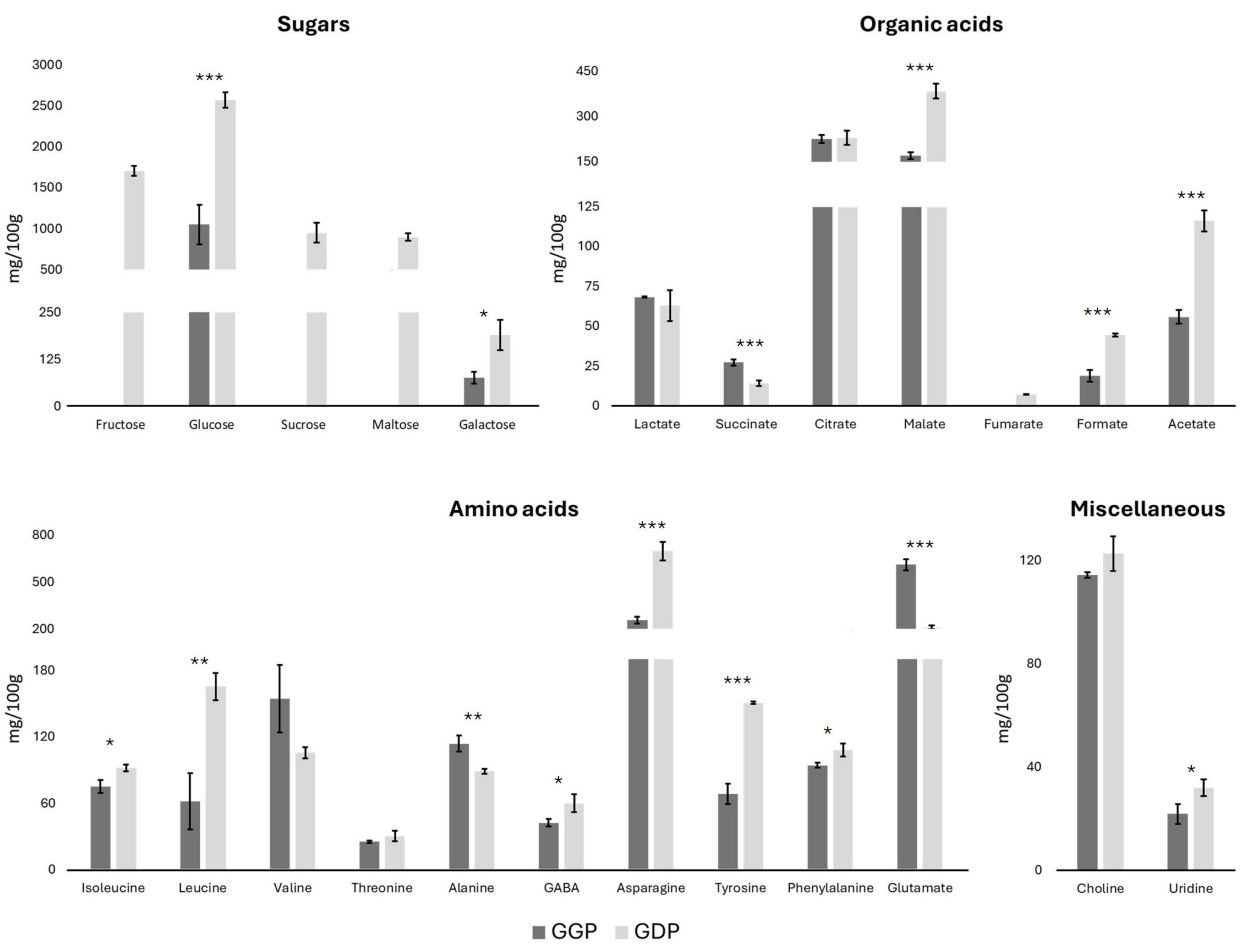
Concentrations of sugars, organic acids, amino acids, choline, and uridine in *G. lutea* flowers, gastric phase (GGP) and duodenal phase (GDP) in mg/100 g of DW ± SD by metabolomics NMR. *p < 0.05; **p < 0.01, ***p < 0.001 significant differences among GGP and GDP.

Moreover, the NMR metabolomics analysis of both GGP and GDP was used to evaluate the amounts of the other metabolites. The NMR spectra are reported in Supplementary Figure S3. In particular, the absolute quantities of five sugars (fructose, glucose, sucrose, maltose, and galactose), seven organic acids (lactate, succinate, citrate, malate, fumarate, formate, and acetate), ten amino acids (isoleucine, leucine, valine, threonine, alanine, GABA, asparagine, tyrosine, phenylalanine, and glutamine), choline, and uridine were determined by internal standard quantification method (Figure 2). Regarding sugars, fructose, sucrose, and maltose were absent in the gastric phase, and arose in the duodenal phase. Glucose and galactose showed a concentration at least two times higher in the duodenal phase with respect to the gastric one, confirming the major sugars release in the duodenal phase. Among organic acids, lactate and citrate quantities were quite constant among the gastric and duodenal phases, while acetate, formate, malate, and fumarate increased their quantity in the duodenal phase by at least two times. On the other hand, succinate decreases its amount from the gastric to the duodenal phase. Regarding the amino acids, isoleucine, valine, threonine, alanine, GABA, and phenylalanine remained relatively constant throughout the gastrointestinal tract progression, while the levels of asparagine, leucine, and tyrosine increased, and the amount of glutamate decreased by a factor of three. Finally, choline and uridine did not vary during the progression through the intestinal tract.

### 3.3 Transepithelial transport study

Caco-2 cells are widely recognized as a reliable *in vitro* model for simulating the human intestinal epithelium. They are extensively used to evaluate the permeability and absorption of drugs, nutrients, and bioactive compounds, providing predictive insights into oral bioavailability (43, 44). For a more realistic simulation of the *in vivo* situation, the combined use of *in vitro* digestion and Caco-2 cell assays provides a more effective approach. In the present study, the basolateral compartment collected after 4 h of exposure was analyzed by HPLC-PDA (Supplementary Figure S4), revealing the following transepithelial transport results: loganic acid 2.63%, swertiamarin 2.92%, gentiopicroside 0.56%, and sweroside 2.35%. The obtained percentages of transport were comparable to those of previous studies on poorly lipophilic molecules, such as anthocyanins (45, 46). However, no studies have been reported in the literature regarding the transepithelial transport of the bioactive molecules investigated here through Caco-2 cells. Although extrapolating *in vitro* findings to the *in vivo* situation remains challenging due to the unknown accumulation of compounds in target tissues, the results obtained with the Caco-2 model were generally consistent with previously published *in vivo* data. In a previous study, Shi et al. reported an oral bioavailability of swertiamarin in Sprague–Dawley rats of 5.6%−7.6% (47). Gentiopicroside, alone and in two decoctions of various Gentiana roots and rhizomes, has been previously studied for oral bioavailability in Wistar rats by Wang et al. (48), who reported a value of 4.71% for gentiopicroside alone and an improved bioavailability for administration as a decoction. Sweroside bioavailability was previously studied in Sprague–Dawley rats, obtaining a value of 11.90% (49). However, the oral bioavailability of loganic acid has not been previously studied. In both systems, swertiamarin and sweroside showed higher uptake than gentiopicroside.

### 3.4 Cell viability

**Figure 3:**
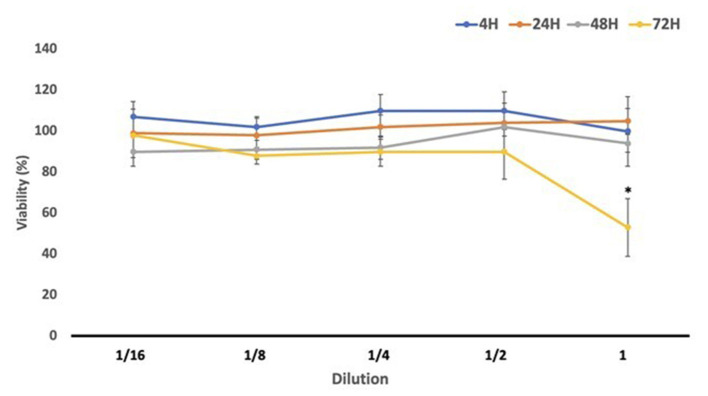
Cell viability (%) results obtained in differentiated Caco-2 cells after exposure to *Gentiana* flower duodenal phase serial dilutions. GDP, *Gentiana* duodenal phase. *p < 0.05 significant differences among the different exposure times.

To assess the cytotoxic potential of *Gentiana lutea* flower extract after digestion, we performed an MTT assay at multiple exposure times (4, 24, 48, and 72 h), as reported in Figure 3. During the shorter exposures (4 and 24 h), cell viability not only remained above 80% across all concentrations but in several cases exceeded 100%, suggesting a stimulatory effect on cellular metabolic activity. This enhanced viability may be attributed to a hormetic response, where low, non-toxic doses of bioactive compounds promote cellular defense mechanisms or metabolic activity (50). Additionally, values higher than the control can be attributed to the rich composition of *G. lutea* flowers in bioactive phytochemicals, which may enhance cell metabolism, growth, and vitality (22). After 48 h, viability remained high (90%−100%), further supporting the non-cytotoxic profile of GDP at this time point. A significant reduction in cell viability (~60%) was observed only after 72 h of exposure to the undiluted GDP, indicating a potential dose- and time-dependent threshold for cytotoxicity. However, all other dilutions at this time point maintained viability levels above 80%, and no statistically significant reduction was observed.

To our knowledge, this is the first study to evaluate the effects of digested *G. lutea* flowers on an *in vitro* gastrointestinal barrier model. The results consistently demonstrated the non-toxic and potentially growth-promoting nature of GDP, which aligns with its known phytochemical richness. Previous studies using *G. lutea* root extracts have also shown high cell viability across various models. For example, Mustafa et al. (51) and Cafaro et al. (52) reported viability values above 100% in PC-12 and SH-SY5Y neuronal cells after 24–48 h exposure to methanolic extracts (25–800 μg/ml). Similarly, Cvetković et al. (53) found no cytotoxicity in MRC-5 lung fibroblasts and Hs294T melanoma cells after 24 h exposure to *G. lutea* root and shoot extracts (0.06–2 mg/ml), with survival rates consistently over 90%. In immune cells, *G. lutea* root aqueous extract (0.1–2 mg/ml) also maintained viability comparable to controls in peripheral blood mononuclear cells (54). Altogether, the mild stimulatory effect observed in our study at lower concentrations and shorter exposures may reflect a hormetic response, whereas the decline in viability with undiluted GDP at prolonged exposure underscores the importance of dosage in balancing safety and efficacy. These findings support the potential application of *G. lutea* flower extract as a safe functional ingredient, with promising implications for the food and nutraceutical industries.

### 3.5 Gene expression analysis

**Figure 4:**
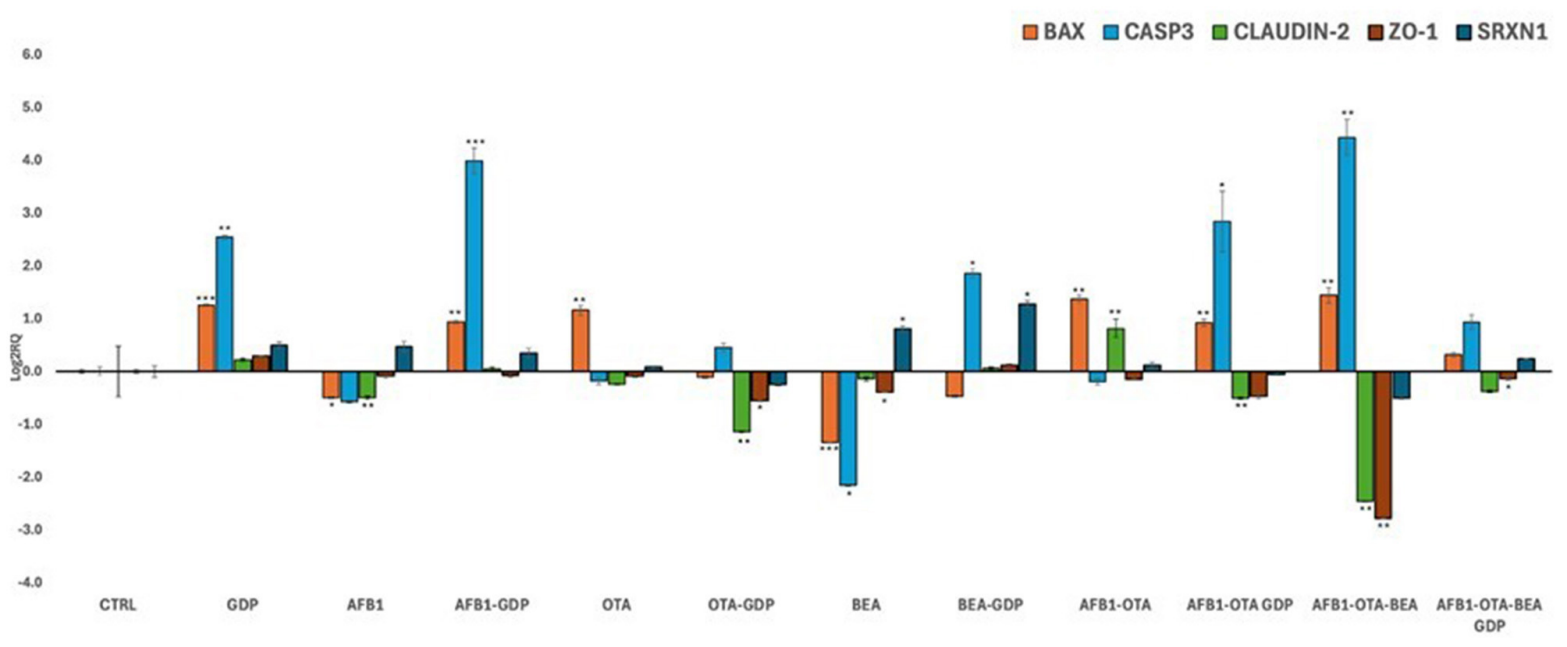
Bar plots showing relative expression of BAX, CASP3, Claudin-2, ZO-1, and SRXN1 when compared to control (Log_2_RQ = 0) after exposure to AFB1, OTA, and BEA individually and in combination among them (100 nM in all cases) or combined with GDP (1:1 as dilution) during 4 h. *p < 0.05; **p < 0.01, ***p < 0.001 is significantly different from the control. RQ, relative quantification; AFB1, aflatoxin B1; OTA, ochratoxin A; BEA, beauvericin; GDP, *Gentiana* duodenal phase; BAX, Bcl-2 Associated X-protein; CASP3, Caspase 3; CL-2, Claudin 2; ZO-1, Zonula occludens; SRXN1, sulfiredoxin 1.

Transcriptional analysis was conducted via qPCR to investigate the mechanistic responses of Caco-2 cells to individual and combined mycotoxin exposures, as well as the modulatory effect of digested *Gentiana lutea* flower extract. A realistic toxicological assessment requires evaluation under co-exposure conditions, as humans and animals are commonly exposed to multiple mycotoxins simultaneously, albeit at low concentrations. Accordingly, EFSA and other regulatory agencies emphasize the importance of mixture toxicity testing to better reproduce real-world exposures (55). The qPCR analysis results are reported in Figure 4.

#### 3.5.1 Apoptotic regulation

BAX and CASP3, two pivotal genes in the intrinsic mitochondrial apoptosis pathway (56), exhibited distinct transcriptional responses. BAX was downregulated following individual exposure to AFB1 and BEA, while OTA, alone and in combination with AFB1 and BEA, significantly upregulated BAX expression. GDP alone moderately increased BAX expression, and its co-treatment with mycotoxins significantly mitigated the toxin-induced upregulation, restoring expression to control levels (*p* < 0.05). CASP3 expression remained largely unchanged under individual mycotoxin exposure but was robustly upregulated (Log_2_RQ >4, *p* < 0.01) by the AFB1-OTA-BEA combination, highlighting a synergistic pro-apoptotic response. Co-treatment with GDP significantly reduced CASP3 overexpression (*p* < 0.05), supporting its role in modulating mitochondrial apoptotic signaling. These findings align with prior studies demonstrating enhanced apoptosis under combined mycotoxin exposure in Caco-2 cells (57). The ability of GDP to suppress BAX and CASP3 activation echoes previous research showing that *G. lutea*-induced cytoprotection in neuronal models (52), suggesting conserved anti-apoptotic potential across systems.

#### 3.5.2 Tight junction protein integrity

Tight junction proteins CLDN2 and ZO-1 are essential to epithelial barrier function. In this study, the AFB1-OTA-BEA combination significantly downregulated the expression of both CLDN2 and ZO-1 (*p* < 0.05), indicating compromised barrier integrity. Importantly, co-treatment with GDP significantly counteracted this downregulation (*p* < 0.05 for both genes), restoring gene expression levels close to control. These changes were statistically significant, underscoring a robust protective effect of GDP on barrier gene expression. By contrast, GDP alone did not significantly alter TJ gene expression compared to the control. CLDN2 forms selective paracellular channels, while ZO-1 anchors these junctional complexes to the actin cytoskeleton. Although these proteins play distinct roles, assessing both provides a more comprehensive understanding of external insults on the gastrointestinal *in vitro* barrier (58). Disruption of these genes compromises epithelial barrier function, increasing susceptibility to luminal toxins. Prior studies have shown that OTA and AFB1 can disrupt TJ gene expression both *in vitro* and *in vivo* (59–61). Here, GDP appears to preserve barrier function by maintaining tight junction gene expression under mycotoxin stress, potentially through stabilization of cytoskeletal anchoring signaling. This is consistent with reported effects of bioactives like lycopene and resveratrol (62, 63) and with evidence of *G. lutea* improving occludin and claudin gene expression in other gut models (64, 65).

#### 3.5.3 Oxidative stress modulation via Nrf2-SRXN1 pathway

SRXN1 is a critical downstream effector of the Nrf2 (nuclear factor erythroid 2-related factor 2) signaling pathway, a master regulator of cellular antioxidant defenses. Upon activation, Nrf2 translocates to the nucleus and upregulates genes encoding detoxifying and antioxidant enzymes, including SRXN1, NQO1, and HO-1. SRXN1 specifically contributes to redox homeostasis by regenerating overoxidized peroxiredoxins and mitigating peroxynitrite damage (66). SRXN1 expression was differentially regulated across treatments. A slight, non-significant increase was observed in most mycotoxin-exposed conditions, but a significant downregulation occurred following AFB1-OTA-BEA co-exposure (*p* < 0.05). Co-treatment with GDP significantly reversed this effect, restoring SRXN1 expression to control levels (*p* < 0.05). Notably, GDP alone also slightly upregulated SRXN1, though this increase was not statistically significant. Comparable findings were reported by previous studies (67, 68), who observed limited SRXN1 modulation in blood-brain barrier models exposed to mycotoxins and antioxidant-rich extracts. While oxidative stress did not appear to be the primary driver of cytotoxicity in this model, GDP may have triggered a subtle antioxidant response via the Nrf2 pathway, contributing to its overall protective effect. Future studies should investigate the capacity of GDP to directly activate Nrf2 and clarify whether SRXN1 upregulation reflects a functional antioxidant defense or an adaptive response to oxidative insult.

#### 3.5.4 Mechanistic integration

Taken together, these transcriptional findings support a multifaceted protective role for GDP in Caco-2 cells exposed to multiple mycotoxins. GDP significantly reversed BAX and CASP3 upregulation, restored tight junction gene expression (CLDN2, ZO-1) under triple mycotoxin stress, and possibly countered SRXN1 downregulation via mechanisms likely involving Nrf2 activation. These effects were statistically significant where noted, reinforcing that GDP cytoprotection is linked to apoptosis, intestinal integrity, and oxidative stress signaling. This supports the relevance of *G. lutea* flower as a candidate for further exploration in dietary or therapeutic strategies to preserve intestinal health under multi-mycotoxin exposure.

## 4 Conclusions

This study offered new insights into the potential of *Gentiana lutea* flowers as a natural protective agent against mycotoxin-induced gastrointestinal damage. The combined use of advanced analytical techniques, including HPLC-PDA and NMR-based metabolomics, allowed for a comprehensive characterization of the flower's bioactive profile and bioaccessibility. The digested extract modulated the expression of apoptosis- and barrier-related genes in a differentiated Caco-2 cell model exposed to AFB1, OTA, and BEA, suggesting potential protective effects at the molecular level. GDP supplementation mitigated the upregulation of pro-apoptotic markers (BAX, CASP3) and prevented the downregulation of tight junction proteins (CL-2, ZO-1). In addition, GDP can play an important antioxidant role by regulating SRXN1 mRNA level, although further analysis assessing the expression of key antioxidant enzymes is needed to confirm this hypothesis. These findings support the use of *Gentiana lutea* flowers as a functional ingredient that can modulate gut barrier responses to food contaminants. Further *in vivo* investigations are necessary to substantiate the bioactivity of the extract and to delineate its mechanism of action within physiologically relevant systems. Future studies should also encompass assessments of intestinal permeability, host–microbiota interactions, and pharmacokinetic profiling to fully characterize its biological effects.

## Data Availability

The raw data supporting the conclusions of this article will be made available by the authors, without undue reservation.
