# Supplementary material for: The protective effect of Gentiana lutea flower against mycotoxin toxicity in a gastrointestinal barrier in vitro model
Source: Front Nutr. 2025 Aug 12;12:1627476. doi: 10.3389/fnut.2025.1627476 (PMC12378322; doi:10.3389/fnut.2025.1627476)
Supplement: Supplementary file 1 [file Data_Sheet_1.docx]

Supplementary Material

## Supplementary Tables

**Table S1.** ^1^H-NMR signals selected for quantification of each metabolite.

| **Metabolite** | **Assignment** | **ppm** |
| --- | --- | --- |
| Isoleucine | δ-CH_3_ | 0.94 |
| Leucine | δ,δ′ -CH_3_ | 0.96 |
| Valine | γ′-CH_3_ | 1.00 |
| Lactate | β-CH_3_ | 1.34 |
| Threonine | γ-CH_3_ | 1.34 |
| Alanine | β-CH_3_ | 1.49 |
| Acetate | α-CH_3_ | 1.93 |
| GABA | α-CH_2_ | 2.30 |
| Glutamate | γ-CH_2_ | 2.36 |
| Succinate | α, β-CH_2_ | 2.42 |
| Citrate | α,γ-CH | 2.54 |
| Asparagine | β-CH_2_ | 2.90 |
| Choline | +N(CH_3_)_3_ | 3.21 |
| D-Fructofuranose |  | 4.13 |
| Sucrose | CH-3 (fructose) | 4.23 |
| Malate | α -CH | 4.30 |
| β-Galactose | CH-1 | 4.60 |
| β-Glucose | CH-1 | 4.67 |
| β -Maltose | CH-1 | 4.68 |
| Uridine | CH-1 (ribose) | 5.93 |
| Fumarate | α, β -HC=CH | 6.53 |
| Tyrosine | CH-2,6 | 7.20 |
| Phenylalanine | CH-4 | 7.38 |
| Formate | HCOOH | 8.47 |

## Supplementary Figures

**
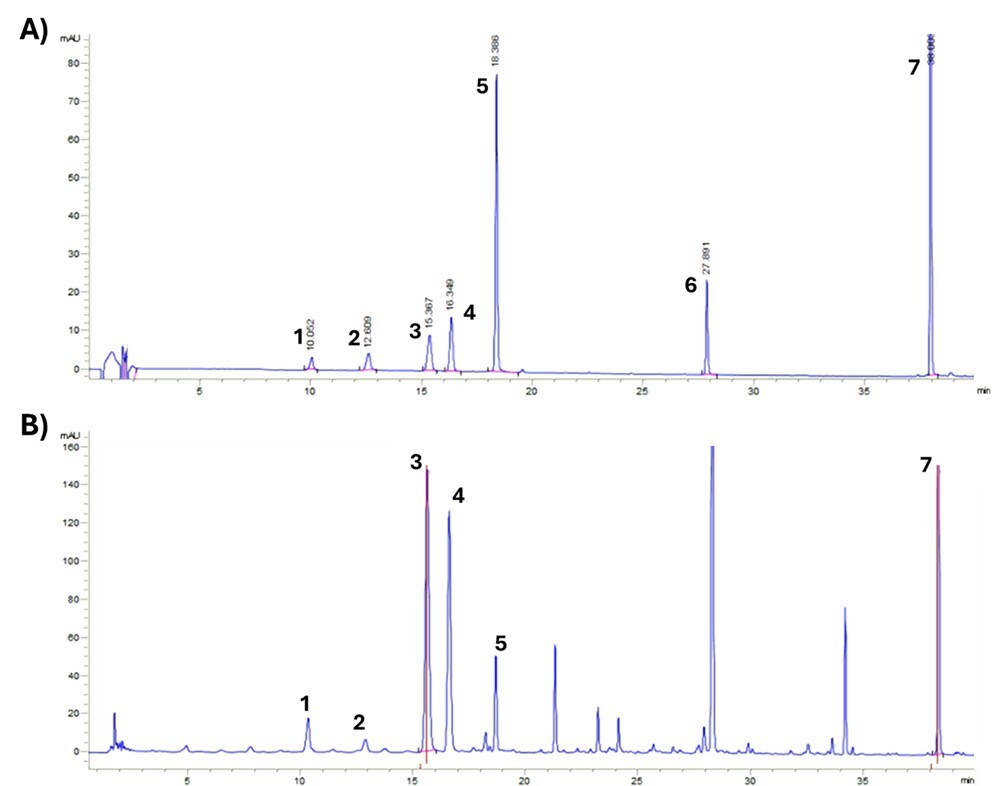
**

**Figure S1.** HPLC chromatograms of standard mix (A) and GME (B) at 258 nm. 1. Loganic acid; 2. Swertiamarin; 3. Gentiopicroside; 4. Sweroside; 5.Mangiferin; 6. Amarogentin; 7. Isogentisin.

**
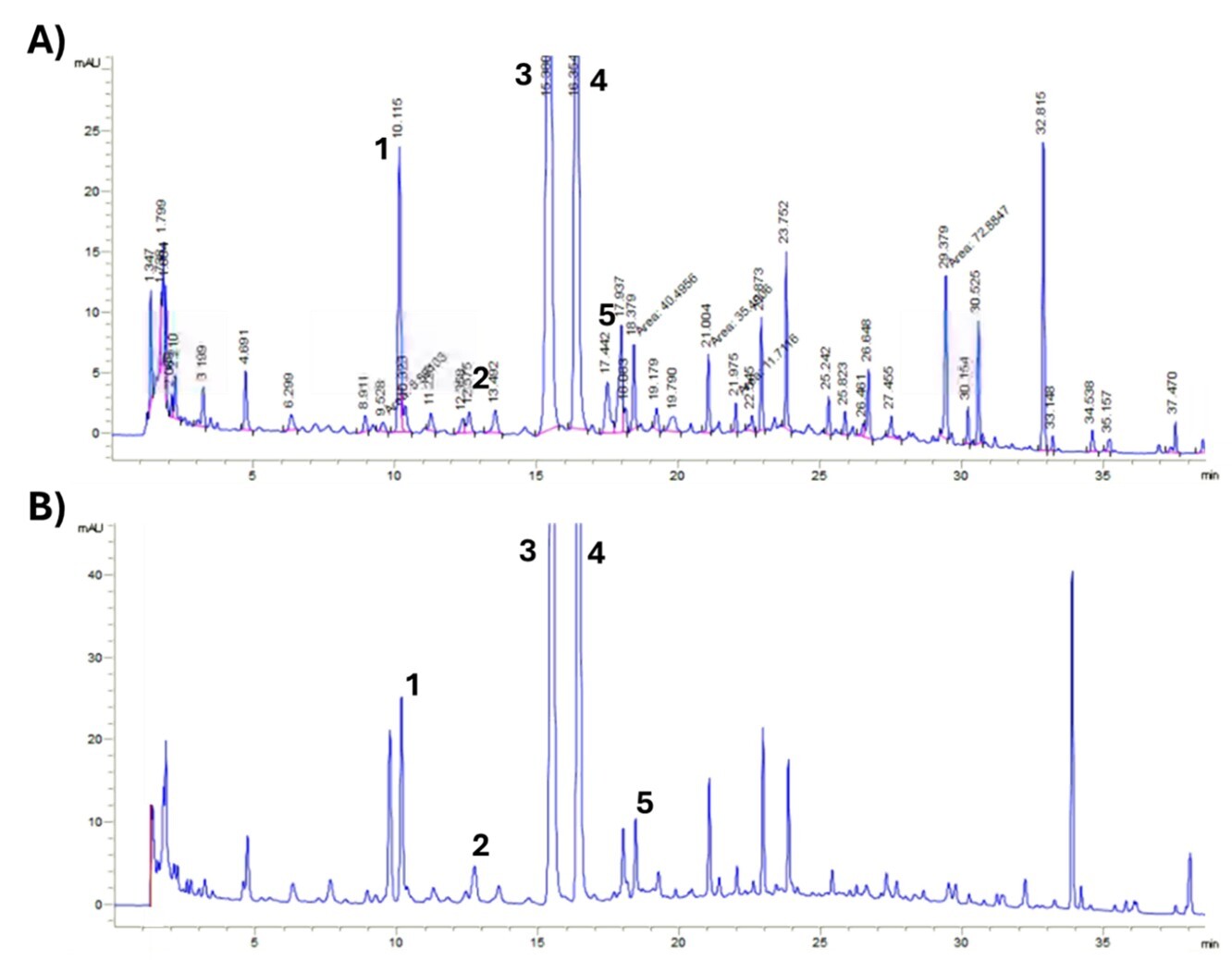
**

**Figure S2.** HPLC chromatograms of GGP (A) and GDP (B) at 258 nm. 1. Loganic acid; 2. Swertiamarin; 3. Gentiopicroside; 4. Sweroside; 5.Mangiferin.

**
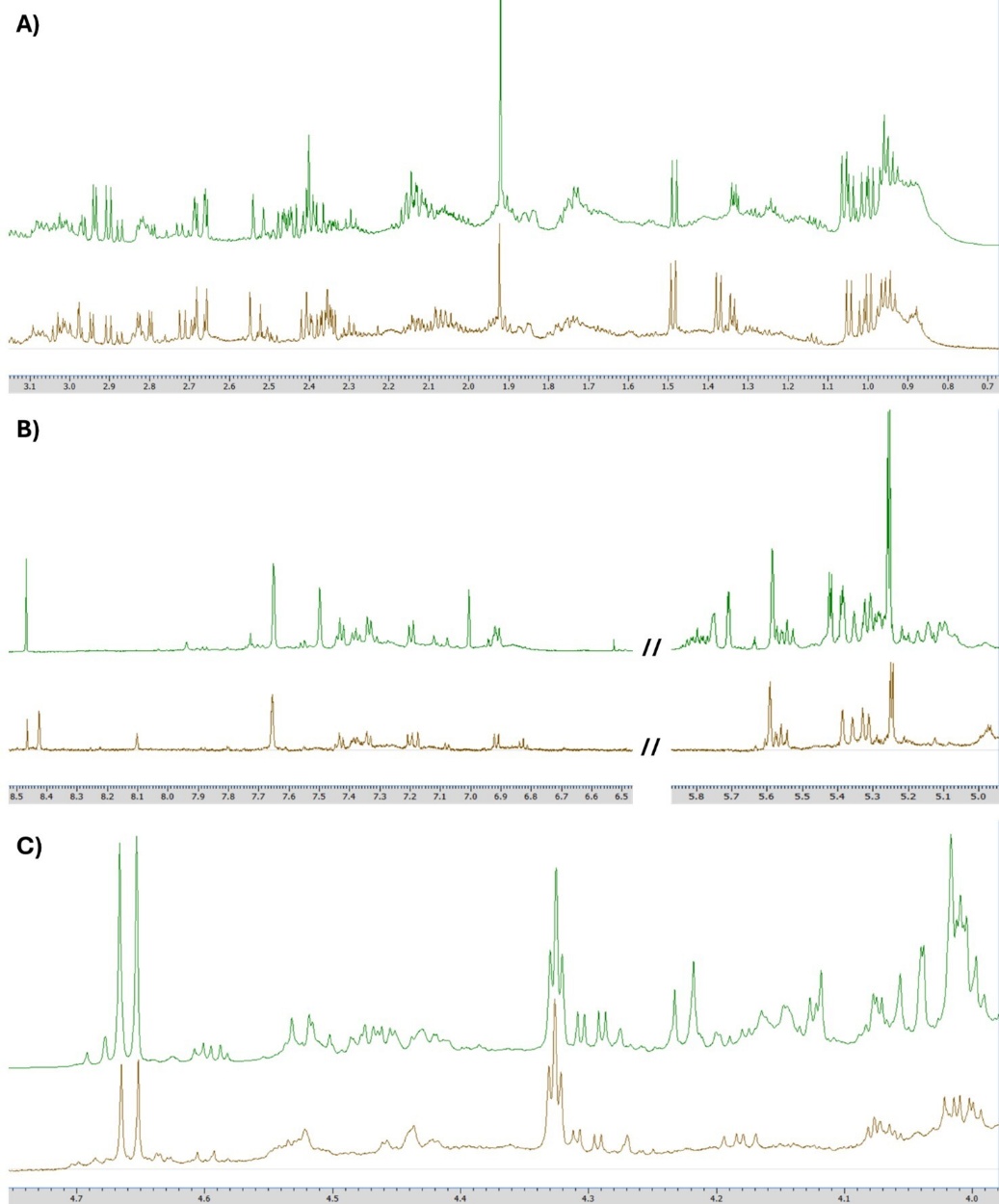
**

**Figure S3.** Overlapped 1H-NMR spectra of GGP (in brown) and GDP (in green). (A) 0.7 – 3.1 ppm region; (B) 5.0 – 8.5 ppm region; (C) 4.0 – 4.7 ppm region.

**
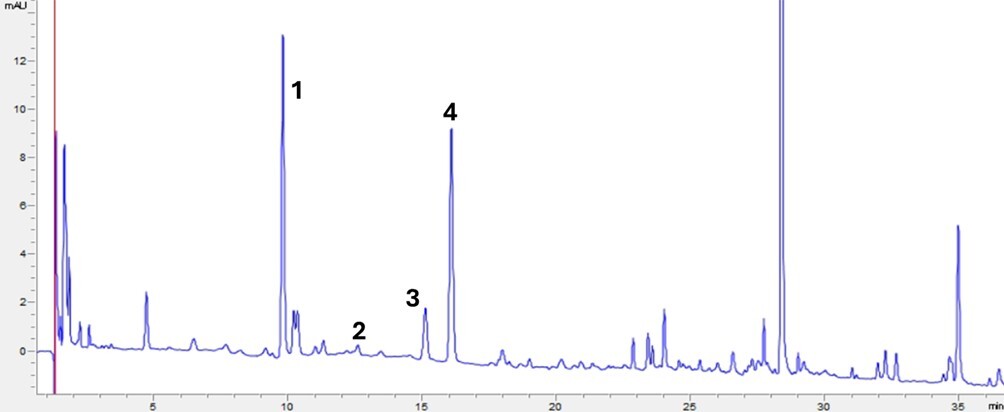
**

**Figure S4.** HPLC chromatograms of basolateral compartment of bioavailability assay at 258 nm. 1. Loganic acid; 2. Swertiamarin; 3. Gentiopicroside; 4. Sweroside.
